# Social distancing stress, anxiety/depression, COVID-19 diagnosis, gender identity, and immigration status

**DOI:** 10.1186/s13690-024-01320-6

**Published:** 2024-06-14

**Authors:** David Adzrago, Jolyna Chiangong, Cameron K. Ormiston, Oluwabunmi M. Dada, Antwan Jones, Faustine Williams

**Affiliations:** 1 Division of Intramural Research, National Institute on Minority Health and Health Disparities, National Institutes of Health, Bethesda, MD USA; 2 Icahn School of Medicine at Mount Sinai, New York, NY USA; 3 Department of Occupational Safety and Health, Murray State University, Murray, KY USA; 4 Department of Sociology, Department of Epidemiology, The George Washington University, Washington, DC USA

**Keywords:** Stress, Social distancing, Mental health, COVID-19, Immigration, Minority

## Abstract

**Background:**

Strict social distancing public health measures to decrease COVID-19 spread increased social distancing stress. However, differences in social distancing stress by anxiety/depression symptoms are understudied, especially based on COVID-19 diagnosis status, gender identity, and immigration status. We examined whether the association between social distancing stress and anxiety/depression symptoms was moderated by COVID-19 diagnosis status, gender identity, and immigration status. We further examined the associations of social distancing stress with anxiety/depression symptoms, gender identity, and immigration status among individuals with and without COVID-19.

**Methods:**

We utilized data from a national cross-sectional survey among adults aged **≥** 18 years in the United States between May 13, 2021, and January 9, 2022 (*n* = 5,255). Multivariable logistic regression models were used to examine the associations.

**Results:**

The prevalence of social distancing stress was higher among individuals with COVID-19 (79.23%) than among those without COVID-19 (67.51%). We observed significant associations between social distancing stress and anxiety/depression symptoms, moderated by COVID-19 diagnosis status, immigration status, and gender identity, respectively. Anxiety/depression symptoms were associated with social distancing stress among both individuals with and without COVID-19. Gender identity and immigration status were associated with social distancing stress among only individuals without COVID-19.

**Conclusions:**

Our findings revealed that the association between social distancing stress and anxiety/depression varied by COVID-19 diagnosis status, gender identity, and immigration status. The findings underscore the need for more targeted psychological distress strategies to reduce social distancing stress and anxiety/depression among diverse US populations, while considering the impacts of COVID-19 diagnosis status, gender identity, and immigration status.

**Table:** 

**Text box 1. Contributions to the literature**
• Limited literature examined social distancing stress (SDS), especially in relation to mental health, COVID-19 diagnosis status, gender identity, and immigrant status.
• People with anxiety/depression symptoms, particularly those with COVID-19, identified as men, and are foreign-born individuals, have more burdens of SDS.
• More targeted psychological distress research and strategies are needed to evaluate and reduce SDS among diverse populations, while considering the impacts of COVID-19 diagnosis status, gender identity, and immigration status.

## Background

According to the Centers for Disease Control and Prevention, over 6 million hospitalizations and over 1 million deaths due to COVID-19 have occurred since the start of the global pandemic [1]. As a strategy to limit COVID-19 transmission, strict public health measures, such as social distancing, were implemented [2]. These strategies prioritized physical health over social and mental health, but emerging literature suggests that social distancing measures were associated with increased acute stress [3–5]. Diminished feelings of social connection created by social distancing have also been linked to depression in adult populations, greater social anxiety, and loneliness [6, 7]. Nearly two in five people in the United States (US) reported adverse mental or behavioral health experiences during the COVID-19 pandemic [8]. However, the use of COVID-19 diagnoses status to examine differences in adverse mental health symptoms, including stress, among different sociodemographic groups is sparse.

People are social beings and, therefore, long-term isolation is likely to contribute to elevated levels of psychological distress directly, and social distancing policies may have also contributed to this distress. However, other life stressors stemming from and exacerbated by the pandemic (e.g., poor post-confinement work-related expectations, job loss, unsafe employment conditions, economic instability) could have also played a significant role in the expression of heightened feelings of loneliness, anxiety, and mental health difficulties [9, 10]. Particularly, little is known about how anxiety and depression impact social distancing stress (SDS). Meta-analytic reviews and theories reported that mental health symptoms, including anxiety and depression, are significant risk factors for stress generation or exposure [11–14]. According to stress generation theory, stress generation is higher among individuals with increased psychopathology or mental health problems than those with lower mental health problems, due to interference of mental health problems in daily and social activities [11–14]. Thus, individuals with elevated mental health problems contribute greater stress generation than their counterparts with lower mental health problems. In the context of SDS, experiencing anxiety and depression may influence the ability to cope with social distancing due to heightened social isolation and decreased social support. Therefore, exploring the influence of mental health symptoms such as anxiety and depression on SDS may be central to understanding the impact of mental health on SDS during the pandemic.

Isolation and larger structural changes may impact sociodemographic groups differently, potentially leading to SDS variations across these groups. For instance, studies found that sexual and gender minority (SGM) individuals reported greater depression symptoms than non-SGM individuals throughout the pandemic [15–17]. This could be because SGM individuals are often subjected to high levels of prejudice and discrimination that could contribute to mental health symptoms, including stress [18, 19]. However, the influence of sexual and gender identity on SDS is unknown. Examining the intersection of sexual and gender identity, COVID-19 diagnosis status, SDS, and anxiety/depression symptoms can help public and mental health professionals and policymakers effectively personalize resources to address stress and its debilitating physical, mental, and behavioral health consequences.

Immigration status is another potential factor that may explain SDS-related disparities in the population. Approximately 79% of all immigrants in the US labor force and 74% of undocumented workers are essential workers, which limits their ability to engage in optimal social distancing practices and increases virus exposure risk [20]. Some immigrants, including those undocumented, utilize the emergency room as their primary care source, given that many undocumented immigrants do not have primary care providers, and this increases COVID-19 exposure risk [20]. Immigration status also impacts the ability to access government-sanctioned financial resources during the pandemic, which may place additional stress on immigrant families. Additionally, the pandemic resulted in substantial decline in minority- and immigrant-owned businesses due to widespread social-distancing restrictions [21]. This could have created additional stress on this population, justifying the need for more research to ascertain immigration status-related disparities in SDS.

While anxiety/depression symptoms, gender identity, and immigration status may play major roles in examining SDS, sociodemographic characteristics such as age, race/ethnicity, level of education completed, annual household income, and health insurance may also explain differences in SDS. Income levels have been shown to influence anxiety/depression symptoms and the ability to practice social distancing [22]. Residents of low-income neighborhoods are less likely to have jobs that allow working from home (e.g., essential workers) compared to high-income neighborhoods, which impacts the ability to socially distance adequately [23]. In addition, racial/ethnic minorities experienced a disproportionate impact of COVID-19 incidence and disease severity even within the first few months of the pandemic [22].

Overall, there is limited literature examining SDS in relation to anxiety, depression, gender identity, and immigrant status, while considering other sociodemographic characteristics. This current study examines the associations between SDS and anxiety/depression symptoms, gender identity, and immigration status based on COVID-19 diagnosis status, while accounting for other sociodemographic characteristics. Specifically, this study aims to: (1) determine the prevalence of SDS by COVID-19 diagnosis status; (2) estimate the prevalence of and differences in SDS by anxiety/depression symptoms, gender identity, immigration status, and other sociodemographic characteristics across COVID-19 diagnosis status; and (3) determine whether COVID-19 diagnosis status, gender identity, or immigration status moderate the association between SDS and anxiety/depression symptoms. The following three research questions were used to address the aims above: (1) Does the prevalence of SDS vary by anxiety/depression symptoms, gender identity, immigration status, and other sociodemographic characteristics among individuals with and without COVID-19 diagnosis? (2) Do gender identity, immigration status, and COVID-19 diagnosis status moderate the association between SDS and anxiety/depression symptoms? (3) Do the associations between SDS and anxiety/depression symptoms, gender identity, and immigration status vary within individuals with and without COVID-19 diagnosis, adjusting for other sociodemographic characteristics (e.g., age, education level)? These findings can help public and mental health professionals develop tailored interventions and policies to address mental health disparities during future public health emergencies.

## Methods

### Procedures and participants

We utilized data from a national cross-sectional survey of adults aged **≥** 18 years in the US using the services of Qualtrics LLC, who recruited and distributed the online survey to the participants in English. Qualtrics used proprietary consumer panels to randomly choose participants with matching demographic characteristics to complete the survey. To ensure and enhance representativeness of the participants, we oversampled low income (<$25,000 annual household income) and rural adults among non-Hispanic White, non-Hispanic Black, Hispanic adults, and foreign-born participants. The self-reported residence in rural area was cross-referenced with zip codes already collected by Qualtrics. The survey was conducted between May 13, 2021, and January 9, 2022, resulting in about 5,938 (59.38% response rate) participants who completed the survey out of 10,000 surveys distributed. The surveys were reviewed by Qualtrics through their expert review fraud detection to detect “bots” and prevent multiple submissions. We attained 5,413 observations after data cleaning by Information Management Services Inc. The data cleaning involves flagging surveys that did not meet the completion rate and the timing criteria: The participants were retained as final sample if they completed 80% or more of 102 survey questions for not less than 5 min. A $5–$10 gift card was used by Qualtrics (based on their survey panel policy) to compensate each participant for completing the survey.

Our current analysis included only the samples (*n* = 5,255) with complete responses to SDS and COVID-19 diagnosis status questions. Ethical approval was obtained for this study (Institutional Review Board [IRB] number: 000308) from the National Institutes of Health IRB. An online informed consent was obtained from the participants.

### Measures

#### Outcome variable

*Social distancing stress.* The participants were asked how stressful social distancing has been for them, and the responses were very stressful, somewhat stressful, a little stressful, or not at all stressful.

#### Explanatory variable

*Anxiety/depression symptoms*. Four questions based on the Patient Health Questionnaire-4 (PHQ-4) scale were used to derive anxiety/depression symptoms among the participants. The participants reported how often they experienced the following symptoms over the last two weeks; (1) feeling nervous, anxious or on edge; (2) not being able to stop or control worrying; (3) feeling down, depressed or hopeless; and (4) little interest or pleasure in doing things [24, 25]. The response options for each of the four questions or items include not at all = 0, several days = 1, more than half the days = 2, or nearly every day = 3. Summation of the responses across the four questions for the PHQ-4 range from 0 to 12, indicating normal (scores = 0–2), mild (scores = 3–5), moderate (scores = 6–8), and severe (scores = 9–12).

### Moderators

*COVID-19 diagnosis status.* Two questions were combined to determine the COVID-19 diagnosis status of the participants. The questions (yes/no response options) were: “Have you been tested for Coronavirus/COVID-19?” and “Was the test for Coronavirus/COVID-19 positive?” Individuals have COVID-19 if they reported testing positive for COVID-19. Otherwise, they were considered to have no COVID-19.

*Gender identity* (gender minority [non-binary/transgender/something else], man, woman) and *immigration status/place of birth* (foreign-born, US-born) were also reported by the participants. Gender identity survey question options, including non-binary, transgender, and something else, were recoded as gender minority in this study due to small samples within the categories of gender identity.

### Covariates

*Sociodemographic characteristics.* The participants reported their age (18–25, 26–34, 35–49, 50–64, 65 or more), race/ethnicity (Asian, Black/African American, Latino/Hispanic, White, and others (African, American Indian or Alaska Native, Middle Eastern or North African, Multiracial, Pacific Islander), level of education completed (less than High School, High School diploma or GED, Some college/vocational or technical school, and college or higher degree), annual household income (less than $25,000, $25,000 to < $35,000, $35,000 to < $50,000, $50,000 to < $75,000, and $75,000 or more), and health insurance (yes/no).

### Statistical analyses

We used Stata/SE version 16 [26] to compute the prevalence of SDS by COVID-19 diagnosis status and present the results in a bar graph. Stratified by COVID-19 diagnosis status, descriptive and bivariate statistics were obtained to describe the prevalence of and differences in SDS by gender identity, immigration status/place of birth, anxiety/depression symptoms, age, race/ethnicity, level of education completed, annual household income, and health insurance (Table 1). The bivariate differences were tested using Pearson Chi-Squared (χ^2^) tests. We also conducted unadjusted moderation analyses to test whether COVID-19 diagnosis status, gender identity, or immigration status moderate the association between SDS and anxiety/depression (Figs. 2, 3 and 4). The moderation analyses were conducted with logistic regression models by first testing the interaction between anxiety/depression and each of the moderators (i.e., COVID-19 diagnosis status, gender identity, and immigration status). We computed the interaction effects (if there was a statistically significant interaction) by estimating the average predicted probabilities using margins command in STATA; the estimates were presented in graphs with marginsplots (Figs. 2, 3 and 4). Before conducting the moderation analyses, we conducted univariate logistic regression analysis to assess the association between SDS and anxiety/depression symptoms. Two multivariable logistic regression models were used to examine the associations between SDS and gender, immigrant status, and anxiety/depression, adjusting for age, race/ethnicity, level of education completed, annual household income, and health insurance (Table 2). The first model evaluated the participants not reporting COVID-19, and the second model examined the participants reporting COVID-19. The Wald chi-squared (χ^2^) method was used to determine the statistical significance of the estimated coefficients. Adjusted odds ratios (AORs) with 95% confidence intervals (CIs) were reported for the logistic regression models. Statistical significance was set at α < 0.05 based on 2-sided hypothesis testing for all statistical tests.

## Results

### Descriptive and bivariate differences in the prevalence of social distancing stress

**Fig. 1:**
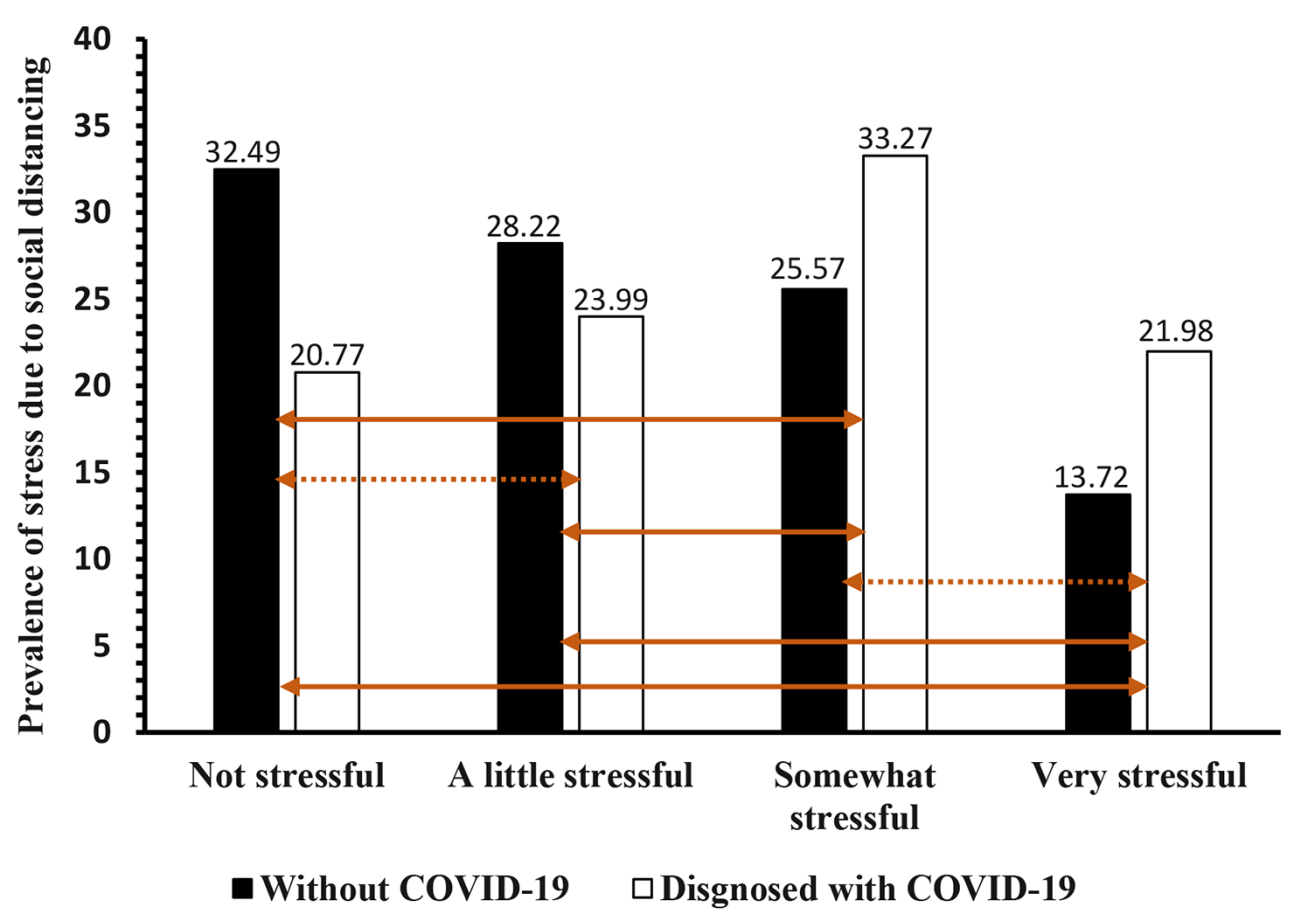
Prevalence of stress due to social distancing by COVID-19 diagnosis status. The orange arrows represent comparisons of each of the six pairs based on Pearson Chi-Squared (χ^2^) tests with Bonferonni adjustment (i.e., the 0.05 alpha level was divided by the total number of pairwise tests to control for type 1 error). The arrows with dashes represent non-statistically significant difference while arrows with solid lines represent statistically significant difference. Thus, significant results were determined at 0.008 alpha level (0.05/6 = 0.008)

Overall, the participants with COVID-19 had a higher prevalence of SDS (79.23%) compared to those without COVID-19 (67.51%) (Table 1); this difference was statistically significant (χ^2^(1) = 28.65, *p* < 0.001). Most of the participants who reported experiencing very stressful or somewhat stressful social distancing were those with COVID-19 (Fig. 1); the difference in the prevalence of SDS categories by COVID-19 diagnosis status was statistically significant (χ^2^(3) = 53.76, *p* < 0.001). However, the pairwise comparison results showed that only the difference in SDS “not being stressful” vs. “a little stressful” or “being somewhat stressful” vs. “very stressful” was not statistically significant based on COVID-19 diagnosis status (Fig. 1).

Bivariate differences in the prevalence of SDS were identified within sociodemographic groups (Table 1). Among the participants without COVID-19, the highest prevalence of SDS was noted among foreign-born persons, those with mild to severe anxiety/depression symptoms, ages 18–25 and 26–34 years old, Latino/Hispanic individuals, had college or higher education, $25,000 or more annual household income, and had health insurance. No differences were observed based on gender identity. Among those with COVID-19, however, the differences were only noted based on anxiety/depression symptoms. Those with mild to severe anxiety/depression symptoms had the highest prevalence of SDS.

**Table 1:** Descriptive and bivariate analyses of stress due to social distancing by sociodemographic characteristics and anxiety/depression stratified by COVID-19 diagnosis status (*n* = 5,255)

	Without COVID-19				Diagnosed with COVID-19		
		Social distancing stress				Social distancing stress	
	Total, *n*	*n* (%)	*p*-value		Total, *n*	*n* (%)	*p*-value
**Overall**	4,759	3,213 (67.51)			496	393 (79.23)	
**Explanatory factors**							
**Anxiety/depression symptoms**			< 0.001				< 0.001
Negative/Normal	2,595 (54.53)	1,503 (57.92)			173 (34.88)	117 (67.63)	
Mild	1,035 (21.75)	804 (77.68)			133 (26.81)	113 (84.96)	
Moderate	609 (12.80)	502 (82.43)			86 (17.34)	75 (87.21)	
Severe	520 (10.93)	404 (77.69)			104 (20.97)	88 (84.62)	
**Moderators**							
**Gender identity**			0.054				0.317
Gender minority	99 (2.08)	59 (59.60)			13 (2.62)	10 (76.92)	
Man	1,667 (35.03)	1,102 (66.11)			186 (37.50)	154 (82.80)	
Woman	2,993 (62.89)	2,052 (68.56)			297 (59.88)	229 (77.10)	
**Immigration status**			0.004				0.085
Foreign-born	1,101 (23.14)	783 (71.12)			100 (20.16)	73 (73.00)	
US-born	3,658 (76.86)	2,430 (66.43)			396 (79.84)	320 (80.81)	
**Covariates**							
**Age groups**			< 0.001				0.415
18–25	647 (13.60)	463 (71.56)			103 (20.77)	83 (80.58)	
26–34	905 (19.02)	653 (72.15)			123 (24.80)	101 (82.11)	
35–49	1,543 (32.42)	1,088 (70.51)			164 (33.06)	131 (79.88)	
50–64	1,162 (24.42)	734 (63.17)			86 (17.34)	65 (75.58)	
65 or older	502 (10.55)	275 (54.78)			20 (4.03)	13 (65.00)	
**Race/ethnicity**			< 0.001				0.844
Asian	522 (10.97)	370 (70.88)			27 (5.44)	21 (77.78)	
Black/African American	1,166 (24.50)	719 (61.66)			121 (24.40)	96 (79.34)	
Latino/Hispanic	842 (17.69)	623 (73.99)			116 (23.39)	96 (82.76)	
White	2,039 (42.85)	1,385 (67.93)			208 (41.94)	162 (77.88)	
Other	190 (3.99)	116 (61.05)			24 (4.84)	18 (75.00)	
**Level of education completed**			0.016				0.451
Less than High School	272 (5.72)	179 (65.81)			30 (6.05)	25 (83.33)	
High School diploma or GED	1,078 (22.65)	696 (64.56)			126 (25.40)	97 (76.98)	
Some college/vocational or technical school	1,547 (32.51)	1,034 (66.84)			157 (31.65)	120 (76.43)	
College or higher degree	1,862 (39.13)	1,304 (70.03)			183 (36.90)	151 (82.51)	
**Annual household income**			0.010				0.061
Less than $25,000	1,187 (24.94)	752 (63.35)			90 (18.15)	63 (70.00)	
$25,000 to < $35,000	711 (14.94)	486 (68.35)			91 (18.35)	76 (83.52)	
$35,000 to < $50,000	735 (15.44)	508 (69.12)			79 (15.93)	67 (84.81)	
$50,000 to < $75,000	882 (18.53)	600 (68.03)			97 (19.56)	81 (83.51)	
$75,000 or more	1,244 (26.14)	867 (69.69)			139 (28.02)	106 (76.26)	
**Health insurance**			0.004				0.089
No	646 (13.57)	404 (62.54)			54 (10.89)	38 (70.37)	
Yes	4,113 (86.43)	2,809 (68.30)			442 (89.11)	355 (80.32)	

### Association between social distancing stress and anxiety/depression: moderation analysis

**Fig. 2:**
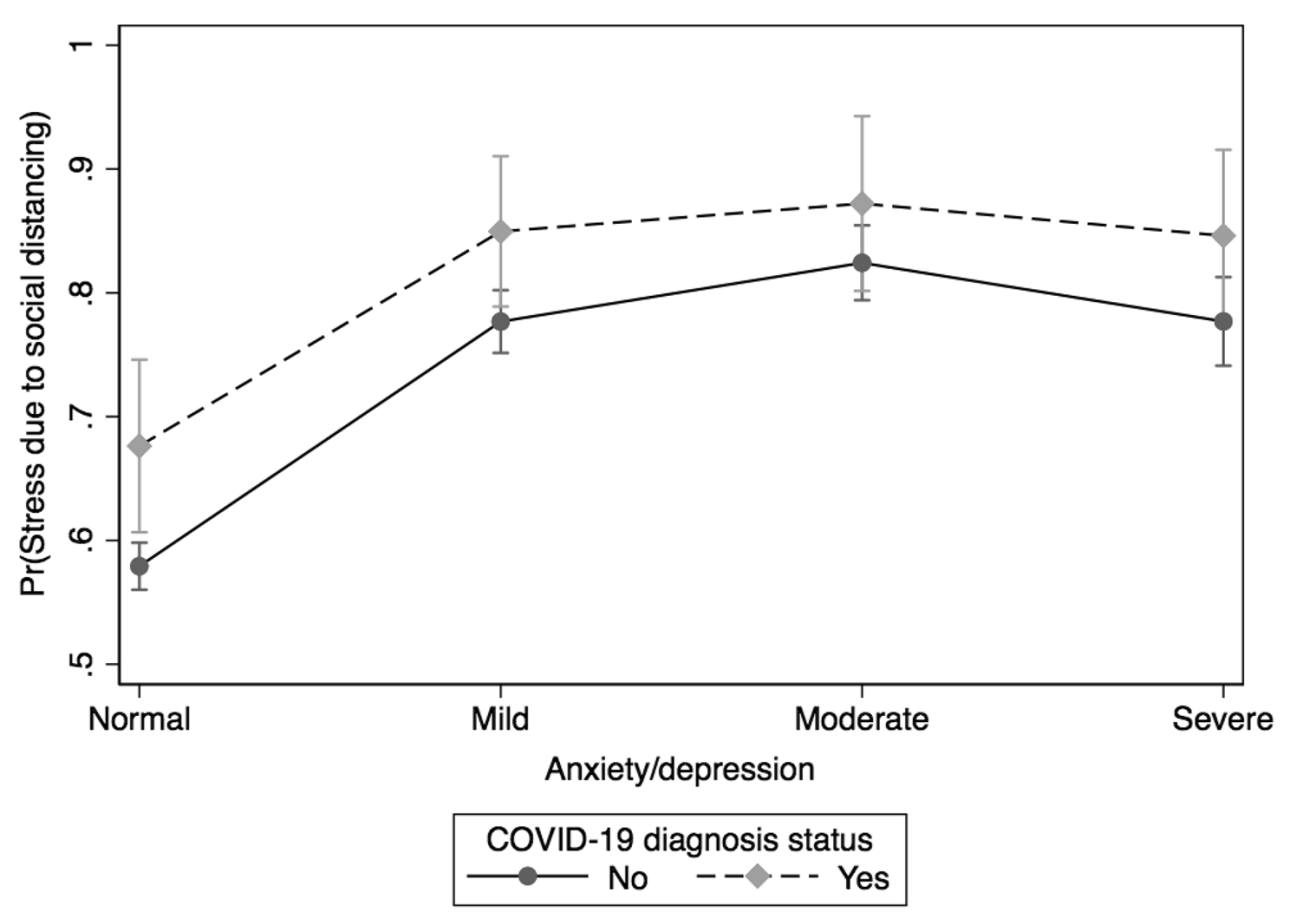
Differences in stress due to social distancing between and within anxiety/depression symptoms and COVID-19 diagnosis status

**Fig. 3:**
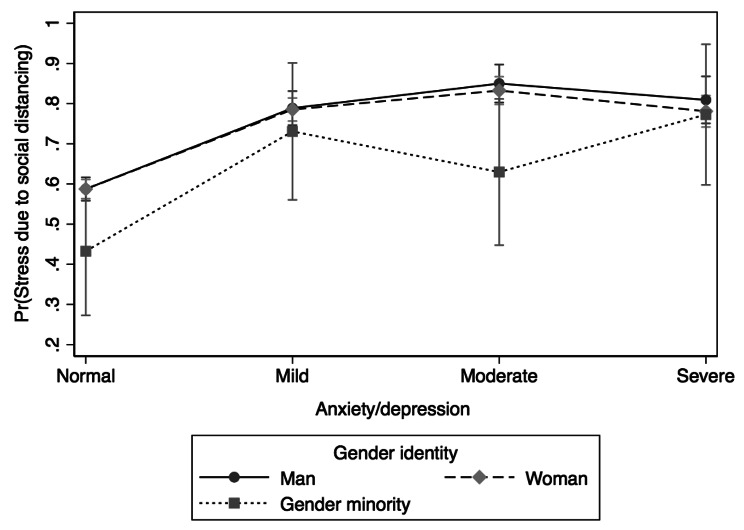
Differences in stress due to social distancing between and within anxiety/depression symptoms and gender identity

**Fig. 4:**
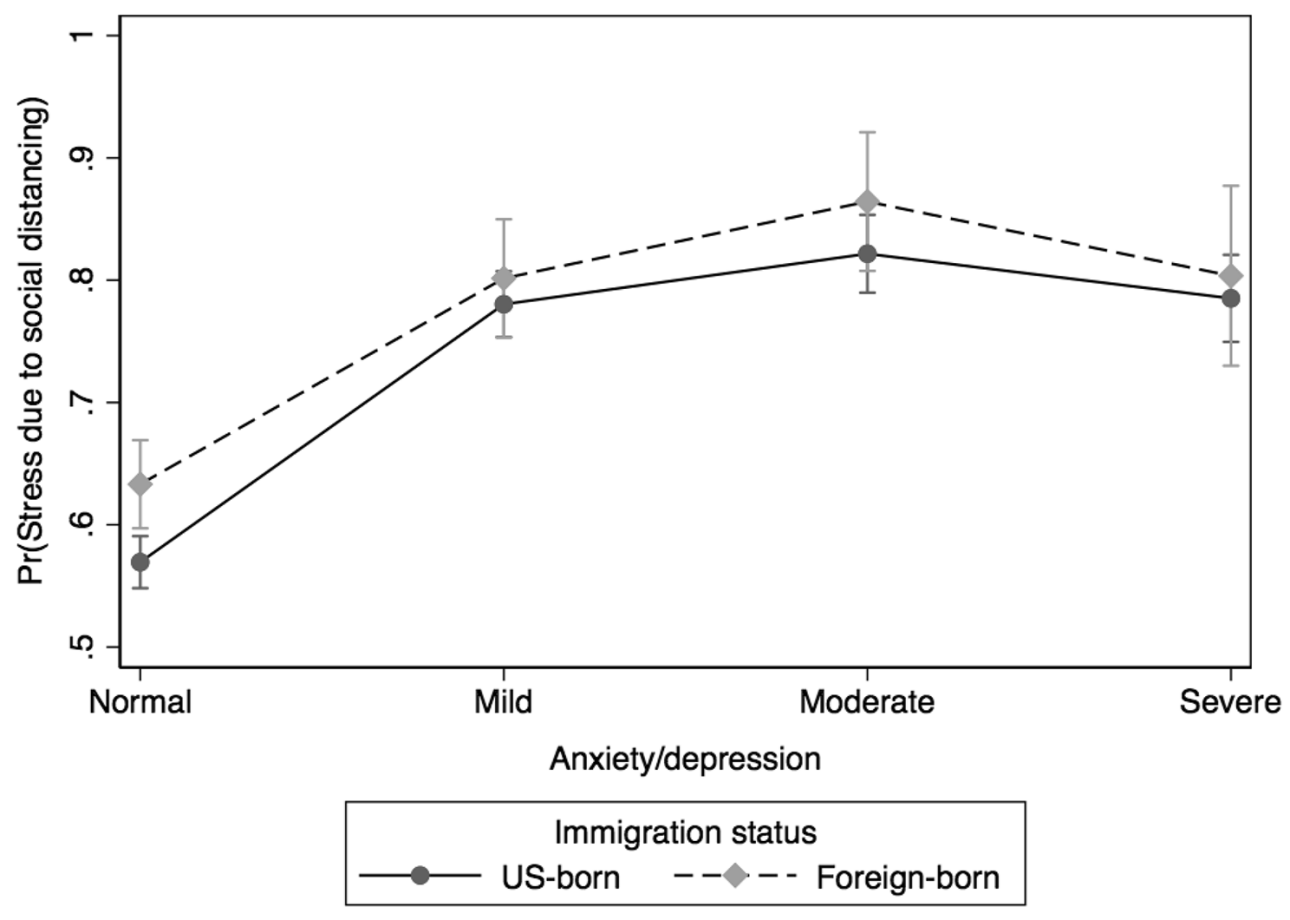
Differences in stress due to social distancing between and within anxiety/depression symptoms and immigration status/place of birth

The univariate logistic regression analysis assessing the association between SDS and anxiety/depression symptoms revealed statistically significant (χ^2^(3) = 289.19, *p* < 0.001) results (results table not shown): individuals with mild (OR = 2.59; 95% CI: 2.21, 3.03), moderate (OR = 3.47; 95% CI: 2.80, 4.28), or severe (OR = 2.64; 95% CI: 2.15, 3.25) anxiety/depression had higher odds of SDS compared to those with negative/normal anxiety/depression symptoms. Moderation effects of COVID-19 diagnosis status, gender identity, and immigration status on the association between SDS and symptoms of anxiety/depression were examined using moderation analysis and marginsplot. The results of the moderation analysis are presented in Figs. 2, 3 and 4.

Figure 2 shows the moderation effects of COVID-19 diagnosis status on the association between SDS and anxiety/depression symptoms. There was a significant interaction between COVID-19 diagnosis status and symptoms of anxiety/depression (χ^2^(7) = 280.91, *p* < 0.001). In general, the participants with symptoms of anxiety/depression and who were diagnosed with COVID-19 had a higher probability of experiencing SDS compared to their counterparts without COVID-19. Within both those diagnosed with COVID-19 and those without, the highest probability was noticed among those with moderate symptoms of anxiety/depression.

Gender identity moderated the association between SDS and symptoms of anxiety/depression (χ^2^(11) = 278.91, *p* < 0.001). As shown in Fig. 3, those who identified as men and experienced moderate anxiety/depression had the highest likelihood of experiencing SDS. Within each gender identity group, the highest likelihoods were noted for men and women with moderate anxiety/depression, while the highest likelihoods were observed within the gender minority with severe symptoms.

The association between SDS and symptoms of anxiety/depression was moderated by immigration status/place of birth (χ^2^(7) = 280.47, *p* < 0.001). Figure 4 displays the subgroup differences in SDS based on anxiety/depression and immigration status/place of birth. Overall, foreign-born individuals with anxiety/depression, especially those with moderate symptoms, had the highest probability of experiencing SDS than their US-born counterparts. The highest likelihood was noticed among foreign-born and US-born groups with moderate anxiety/depression symptoms.

### Social distancing stress and its associated factors: multivariable logistic regression analysis

Table 2 presents the results of the adjusted multivariable logistic regression analyses stratified by COVID-19 diagnosis status. Among the participants without COVID-19, gender minority individuals had lower odds of experiencing SDS (AOR = 0.53; 95% CI: 0.34, 0.82) relative to those who identified as a woman. Being born outside the US (vs. US-born) (AOR = 1.28; 95% CI: 1.08, 1.52) and having mild (AOR = 2.63; 95% CI: 2.21, 3.12), moderate (AOR = 3.59; 95% CI: 2.84, 4.52), or severe (AOR = 2.68; 95% CI: 2.13, 3.37) anxiety/depression (vs. negative/normal) were associated with higher odds of experiencing SDS. Lower odds of experiencing SDS were noted among those aged 65 years or more (AOR = 0.63; 95% CI: 0.48, 0.82) compared to those aged 18–25 years; Black/African American individuals (AOR = 0.72; 95% CI: 0.61, 0.84) and other racial/ethnic individuals (AOR = 0.68; 95% CI: 0.50, 0.95) were correlated with lower odds of SDS, relative to White individuals. Having an annual household income (vs. less than $25,000) of $35,000 to < $50,000 (AOR = 1.26; 95% CI: 1.02, 1.56) and $75,000 or more (AOR = 1.38; 95% CI: 1.13, 1.70) and health insurance (vs. no insurance) (AOR = 1.40; 95% CI: 1.16, 1.70) were associated higher odds of SDS.

Only anxiety/depression and annual household income were associated with SDS among the participants with COVID-19. Compared to having no/negative anxiety/depression symptoms, having mild (AOR = 2.83; 95% CI: 1.55, 5.18), moderate (AOR = 3.23; 95% CI: 1.50, 6.95), or severe (AOR = 2.44; 95% CI: 1.24, 4.79) symptoms was associated with higher odds of SDS. These higher odds were also found among those with an annual household income of $25,000 to < $35,000 000 (AOR = 2.15; 95% CI: 1.01, 4.56) and $35,000 to < $50,000 (AOR = 2.55; 95% CI: 1.12, 5.80) compared to those with less than $25,000.

**Table 2:** Multivariable logistic regression analysis of the associations between stress due to social distancing and sociodemographic characteristics and anxiety/depression stratified by COVID-19 diagnosis status

	Without COVID-19			Diagnosed with COVID-19	
	Social distancing stress			Social distancing stress	
	AOR	95% CI		AOR	95% CI
**Explanatory factor**					
**Anxiety/depression symptoms**					
Negative/Normal	Ref	-		Ref	-
Mild	2.63***	(2.21, 3.12)		2.83**	(1.55, 5.18)
Moderate	3.59***	(2.84, 4.52)		3.23**	(1.50, 6.95)
Severe	2.68***	(2.13, 3.37)		2.44*	(1.24, 4.79)
**Moderators**					
**Gender identity**					
Gender minority	0.53**	(0.34, 0.82)		0.82	(0.19, 3.46)
Man	0.96	(0.84, 1.10)		1.43	(0.85, 2.39)
Woman	Ref	-		Ref	-
**Immigration status**					
Foreign-born	1.28**	(1.08, 1.52)		0.57	(0.31, 1.0504)
US-born	Ref	-		Ref	-
**Covariates**					
**Age groups**					
18–25	Ref	-		Ref	-
26–34	1.05	(0.83, 1.33)		1.15	(0.56, 2.36)
35–49	1.02	(0.82, 1.28)		1.07	(0.54, 2.11)
50–64	0.80	(0.64, 1.01)		1.10	(0.50, 2.41)
65 or older	0.63**	(0.48, 0.82)		0.61	(0.19, 1.91)
**Race/ethnicity**					
Asian	0.95	(0.75, 1.20)		1.20	(0.41, 3.51)
Black/African American	0.72***	(0.61, 0.84)		0.92	(0.50, 1.67)
Latino/Hispanic	1.18	(0.97, 1.44)		1.49	(0.76, 2.90)
White	Ref	-		Ref	-
Other	0.68*	(0.50, 0.95)		0.75	(0.26, 2.15)
**Level of education completed**					
Less than High School	Ref	-		Ref	-
High School diploma or GED	0.99	(0.74, 1.34)		0.76	(0.24, 2.39)
Some college/vocational or technical school	1.09	(0.81, 1.47)		0.72	(0.23, 2.23)
College or higher degree	1.24	(0.92, 1.68)		1.16	(0.36, 3.76)
**Annual household income**					
Less than $25,000					
$25,000 to < $35,000	1.22	(0.99, 1.51)		2.15*	(1.01, 4.56)
$35,000 to < $50,000	1.26*	(1.02, 1.56)		2.55*	(1.12, 5.80)
$50,000 to < $75,000	1.21	(0.99, 1.48)		1.93	(0.90, 4.15)
$75,000 or more	1.38**	(1.13, 1.70)		1.08	(0.53, 2.20)
**Health insurance**					
No	Ref	-		Ref	-
Yes	1.40***	(1.16, 1.70)		1.71	(0.85, 3.44)

AOR = Adjusted odds ratio. 95% CI = 95% confidence interval. Ref = Reference group

*p < 0.05, **p < 0.01, ***p < 0.001

## Discussion

This study used COVID-19 status to examine the associations between SDS and mental health disorder symptoms, gender identity, and immigration status, while adjusting for sociodemographic characteristics. To our knowledge, this is the first national survey to examine SDS and anxiety/depression symptoms using COVID-19 diagnoses status. The findings show that SDS varied based on COVID-19 diagnosis status, anxiety/depression symptoms, gender identity, and immigration status. We found that participants diagnosed with COVID-19 (79.23%) had a higher prevalence of SDS than those without COVID-19 (67.51%). Furthermore, foreign-born or immigrant persons, participants with mild to severe anxiety/depression symptoms, and Latino/Hispanic individuals had the highest prevalence of SDS. Particularly, foreign-born individuals with anxiety/depression (especially moderate symptoms) had the highest probability of experiencing SDS than their US-born counterparts. Conversely, gender minority individuals without COVID-19 were less likely to experience SDS compared with those who identified as a woman. These observed associations may be partly due to people diagnosed with COVID-19 being encouraged to engage in stricter protective measures post-diagnosis [27, 28]. Consequently, fear of viral spread, loneliness, and isolation while experiencing symptoms and uncertainties regarding prognosis may be underlying mechanisms for anxiety/depression and higher SDS. The findings underscore the need for more targeted psychological distress strategies to reduce SDS and anxiety/depression among diverse US populations, while considering the impacts of COVID-19 diagnosis status, gender identity, and immigration status.

Our findings are consistent with studies that found that Hispanic individuals [29, 30] and foreign-born persons faced psychological and physical stress from COVID-19 [31–33]. In our study, among study participants without COVID-19, the highest prevalence of SDS was noted among foreign-born persons, young adults (26–34 years), and Latino/Hispanic individuals. Specifically, some of the highest prevalence of SDS was noted among foreign-born populations not reporting COVID-19. Being foreign-born and having mild, moderate, or severe anxiety/depression was associated with higher odds of SDS. Foreign-born populations experience an exacerbation of stressors, which may increase SDS. Immigrant or foreign-born individuals often live in multigenerational homes or are more likely to be in overcrowded homes with insufficient space for social distancing or self-isolation [34–36]. This is influenced by a lack of economic resources and may increase stress due to a decreased ability to adequately social distance. Furthermore, for some immigrant populations, there are barriers to healthcare accessibility and employment limitations. As the pandemic began, government-funded relief programs were initiated to assist with pandemic-induced burdens. Despite these resources, some immigrants lacked qualifications due to their immigration status [20]. For example, the Coronavirus Aid, Relief, and Economic Security Act prohibited most US taxpaying, mixed-status families—families with both undocumented and US citizen members—from obtaining financial relief due to ineligibility [20]. The disproportionate immigrant population in the US labor workforce who are essential workers can also increase COVID-19 exposure and decrease social distancing abilities [20]. Living within high COVID-19 risk areas and being an essential worker can create additional fear and stress of spreading the virus to loved ones due to limited social distancing capability [37]. The combination of these factors may have increased SDS among foreign-born populations.

Some of the highest SDS rates among participants without COVID-19 were observed among Latino/Hispanic individuals. Hispanic/Latino individuals in the US disproportionally make up essential workers [38]. When considering immigration status and race/ethnicity, Hispanic immigrants are also more likely to be essential workers and are less likely to have occupations that allow for virtual, at-home employment, thereby increasing infection risk and, subsequently, disease anxiety [39–41].

Among the participants without COVID-19, the youngest age group had the highest prevalence of SDS. Lower odds of experiencing SDS were noted among those aged **≥** 65 years compared to those aged 18–25 years. These findings are consistent with studies that revealed that younger age groups were more vulnerable to stress, depression, and anxiety symptoms during the pandemic [42, 43]. Factors such as loneliness due to social distancing have served as mediation variables between stress and depression [42]. Social isolation and loneliness may exacerbate stress responses among elderly individuals, and contribute to anxiety, depression, and post-traumatic stress disorder; however, older age groups have also been shown to have higher resilience compared to younger age groups (i.e., 18–34 years old) [43–47]. These differences highlight a need to pay greater attention to SDS in younger age groups, and specifically to unique factors affecting these groups, such as transitions within school/academia (e.g., online learning) and resilience coping [42]. For many students, the shift to remote learning has led to increased social isolation due to separation from teachers, classmates, and friends [48, 49]. Additionally, mental health symptoms such as anxiety and depression were already concerns for younger age groups or populations such as college students [48, 50, 51]. This situation could exacerbate SDS, especially with decreased social connectedness and emotional support. Remote learning can present additional stressors due to sub-optimal physical learning environments [52]. Therefore, considering the influence of factors such as altered academic learning environments is important, as hybrid forms of learning and interactions continue to be present as the influence of COVID-19 evolves.

Moderation effects of COVID-19 diagnosis status, gender identity, and immigration status on the associations between SDS and symptoms of anxiety/depression were noted, suggesting the effects of complex, multi-faceted and multi-layered risk factors for SDS. Implementation of complex, multi-faceted and multi-layered SDS interventions and longitudinal studies should consider the impact of the intersectionality of mental health status, COVID-19 diagnosis status, gender identity, and immigration status. The moderation effects of COVID-19 diagnosis status revealed that those with COVID-19 and anxiety/depression symptoms experienced greater SDS. Our results are consistent with another study that revealed that COVID-19 experiences were associated with greater odds of anxiety/depression diagnoses [53]. Research has also shown that SGM individuals reported greater depression symptoms than non-SGM individuals throughout the pandemic [15]. Notably before the pandemic, gender minority communities experienced significant mental health challenges, which can be attributed to factors such as stigmatization, victimization, discrimination, and barriers to accessing healthcare services [54, 55]. In addition, this community experiences the risk of family rejection which can in turn limit support systems and exacerbate social inequalities (i.e., food insecurity, homelessness, foster care, other unstable housing, poverty), which could adversely affect their mental health and well-being during the current pandemic [55–57]. Contrary to these previous studies, our findings show that the prevalence and odds of SDS are lowest among gender minority individuals compared to those who identified as a man or woman. It is possible that while gender minority individuals generally have high burdens of mental health disorder symptoms, their symptoms may not be significantly impacted by the pandemic compared to their non-gender minority counterparts. Because gender minority individuals may be used to social isolation, stigmatization, victimization, and discrimination, the social distancing may not affect them significantly like their non-gender minority counterparts who may not be used to social isolation or discrimination.

Consequently, SDS may be worsened by heightened depression symptoms and limitations on community interactions and support. Those who identified as men and reported moderate anxiety/depression had the highest likelihood of experiencing SDS. These findings replicate previous studies that reported that men experienced heightened depression, anxiety, and stress due to deterioration of economic status compared to women [58, 59]. Men tend to have fewer friendships than women, and those friendships tend to be activity-based which can be limited during the pandemic [59]. Also, men sometimes experience difficulties confiding in and establishing close social connections with other men, which can contribute to depression, anxiety, and loneliness and be exacerbated due to the social distancing needs during the pandemic [59]. More studies are needed to understand better the pathways linking risk factors to SDS and overall stress experienced by men.

There are limitations to this study. First, given that cross-sectional data were used, no causal relationships can be drawn from our findings. Hence, no causal relationships were established between anxiety/depression symptoms, gender identity, immigration status, Covid-19 diagnosis, and SDS. Also, the measures were self-reported, which are susceptible to social desirability and self-reported biases leading to over or underestimation of health behaviors or outcomes. Further, it is important to note that the variables examined within this study are not comprehensive of all factors that might have contributed to SDS during the Covid-19 pandemic. Hence, there could be residual confounding factors (e.g., household relationships, having children, and variations in social distancing policies) that might affect our findings in this study. Additionally, we computed unweighted analyses (i.e., no survey weight available and applied to the analyses) and therefore the results are not generalizable and representative of the entire US adult population.

## Conclusions

The present study found significant associations between increased SDS and COVID-19 diagnosis, anxiety/depression, and immigration status. Sociodemographic factors such as racial/ethnic background, age, and education levels were associated with SDS. Heightened SDS among individuals with COVID-19 and anxiety/depression indicate a need for additional knowledge about resources to reduce SDS among these populations. The higher prevalence of SDS among immigrants highlight a potential disparity in SDS based on stressors from the COVID-19 pandemic. The potential future implications of our findings are significant and multifaceted. First, the findings emphasize the need for effective allocation of healthcare resources to ensure that healthcare providers develop tailored interventions and services to individuals greatly impacted by the COVID-19 pandemic and SDS. Second, public health interventions may need to ensure that support services and resources are available, accessible, and culturally appropriate for diverse populations. Third, mental health professionals and community organizations may need to work together and develop evidence-based strategies on how individuals can cope effectively with stress due to social distancing. Future research and policy initiatives should further consider and investigate health disparities and inequities in access to mental health support services, to understand the unique experiences of immigrant populations with mental health in the US. Ultimately, our findings contribute to the growing literature examining SDS while determining intersectional sociodemographic characteristics that impact levels of SDS, thereby helping to generate research for more tailored SDS interventions.

## Data Availability

The datasets used and/or analyzed during the current study are available from the corresponding authors on reasonable request.
